# Highly Sensitive Optical Time-Domain Reflectometry: Detecting 0.01 dB Leakage over 1000 km for Classical and Quantum Communication

**DOI:** 10.3390/s25051407

**Published:** 2025-02-26

**Authors:** Michael Yarovikov, Alexander Smirnov, Aziz Aliev, Daniel Strizhak

**Affiliations:** Terra Quantum AG, Kornhausstrasse 25, CH-9000 St. Gallen, Switzerland; asm@terraquantum.swiss (A.S.); aa@terraquantum.swiss (A.A.); dst@terraquantum.swiss (D.S.)

**Keywords:** OTDR, quantum communication, optical fiber

## Abstract

Classical and quantum fiber-optic communication channels are vulnerable to possible intrusions. Quantum key distribution (QKD) provides a physically fundamental and theoretically secure communication method, but the key rate decays over long distances. Recently proposed by our group, a quantum communication protocol based on the physical estimation of an eavesdropper’s influence shows extreme efficiency even at distances of thousands of kilometers. In this paper, we investigate the physical limits of eavesdropper detection using optical time domain reflectometry (OTDR) and demonstrate the successful detection of a 0.01 dB leakage over a distance of 1009 km.

## 1. Introduction

In recent years, quantum cryptographic protocols, whose security is based on the fundamental laws of quantum physics, have become increasingly popular for solving the problem of secure key distribution. This field, known as quantum communication, has been actively developed recently [1]. In quantum communication, it is typically assumed that all information lost in the process is fully accessible to the eavesdropper (Eve), and the evaluation of its influence in the key distribution procedure is made by analyzing the statistical properties of the transmitted bit sequence. Thus, the presence of natural losses significantly limits the maximum distance over which one-way key distribution is possible. This limitation is also known as the PLOB bound [2]. As a result, most experimental implementations show high efficiency over distances not exceeding hundreds of kilometers [3,4,5,6,7,8]. And only a few protocols are able to reach 1000 km [9].

Quantum repeaters [10] are used as an option to increase the distance, but this approach is technically difficult to implement, as the ability to manipulate delicate entangled states is severely limited at the current state of the art. Another original approach to increasing the maximum distance is to use trusted nodes along the transmission line [11], which provides security but with additional requirements.

However, as shown in ref. [12], only local leaks provide useful information to the eavesdropper, not the radiation scattered along the fiber. Using physical-level monitoring, this work predicts a significant improvement in the secret key generation rate. Several subsequent studies follow this approach [13,14], although unfortunately they do not cite the original work. In addition, the ability to estimate the fraction of information intercepted by the eavesdropper can also improve conventional QKD protocols [15]. In addition to QKD, there is also quantum secure direct communication, where secure information is transmitted directly [16,17,18,19]. Recently, a comprehensive quantum communication protocol has been proposed in which the monitoring of the eavesdropper’s information fraction plays a crucial role [12,20]. This monitoring capability enables high key rates to be achieved over distances of hundreds of kilometers, even when optical amplifiers are used. Therefore, the task of high-precision eavesdropping in long-distance fiber links remains a topic of considerable interest.

One of the most convenient approaches to tracking communication fibers is optical reflectometry. It is based on sending light probe pulses into the fiber and processing the returned radiation, which carries information about the attenuation along the fiber and the locations of various local events. Optical time domain reflectometry (OTDR) [21] is a well-established technology for the rough inspection of modern fiber optic telecommunications systems. In recent decades, a number of advanced long-range reflectometry techniques have been developed. Phase-sensitive reflectometry (ϕ-OTDR) [22,23,24,25], coherent reflectometry (C-OTDR) [26,27,28,29,30], chaotic source reflectometry (CC-OTDR) [31] and a number of methods to improve the resolution and monitoring performance of OTDR using various processing algorithms and neural networks [32,33,34,35,36] have been proposed. Although great progress has been made in the above methods of optical line inspection, these methods mainly serve a different purpose. Current ϕ-OTDR solutions can monitor the fiber-optic line and the environment at distances up to 300 km in real time, but they do not detect leaks with high accuracy. The C-OTDR approach can be implemented over distances of approximately one thousand kilometers, but this approach is very expensive to implement and is not well suited for widespread use. The maximum distance of CC-OTDR is 182 km and its main feature is the observation of reflections in the fiber-optic line with extreme resolution. These approaches can achieve large observation distances, but further scaling them with amplifiers is much more challenging than for conventional reflectometry. Thus, standard OTDR still has great research potential for solving long-range high-precision monitoring problems.

In this paper, we demonstrate an experimental implementation of an OTDR-based monitoring approach for long-haul optical fiber links. This approach works well with bidirectional amplifiers, allowing for distances exceeding hundreds of kilometers, and is compatible with both classical systems and the recently proposed control-based quantum key distribution (QCKD) protocol [12,20]. Using a custom reflectometry system and mathematical processing, we report the successful detection of local losses equal to 0.01 dB at a distance of 1009 km with a spatial resolution of 80 m.

## 2. Monitoring Concept

In this section, we describe our monitoring approach and its implementation in the key distribution scenario. The legitimate users Alice and Bob perform a procedure to monitor the current state of the fiber between key distribution sessions. As a result of the monitoring procedure, they extract additional information about possible intrusions. This could be the probability of Eve’s presence, a number of leaks exceeding a threshold, or a single most valuable leak on the line, whatever a control-based key distribution protocol requires. These monitoring estimates can be used in the post-selection and privacy amplification phases to refine the bounds on Eve’s information, leading to adjustments in the key rate or termination of transmission.

In this paper, we follow the approach described in ref. [12]. As a result of the monitoring procedure, we consider the value of $r_{E}$ introduced there, which corresponds to the upper limit of the stolen fraction of the original signal. We also rely on a conventional reflectometry technique, which we consider to be a promising approach to achieve distances of hundreds of kilometers [15]. In modern optical telecommunication links, the propagation of information pulses over distances of hundreds and thousands of kilometers is commonly achieved by using optical amplifiers based on erbium-doped fiber (EDFA) [37]. The amplifiers with the same gain factors are typically installed at an equal distance from each other to compensate for the natural losses in a fiber. In our experiments, we follow the previous work [20] and use the channel design for our 1020 km fiber line with 10 dB amplifiers installed every 50 km. This design is experimentally confirmed and suitable for monitoring-based quantum key distribution and is compatible with line monitoring via OTDR. To support forward transmission of the OTDR probe pulses and reverse transmission of the scattered light, all amplifiers in our fiber line have been designed to be bidirectional. In addition, amplifiers have the same gain factor for both strong probing pulses of tens of milliwatts of power and weak backscatter of nanowatts.

### 2.1. OTDR Operation

**Figure 1:**
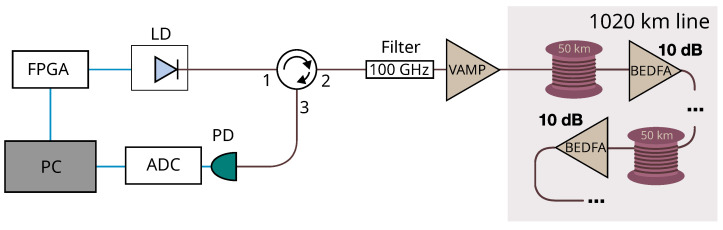
Schematic of the custom OTDR device. LD—laser diode; FPGA—field-programmable gate array; VAMP—variable bidirectional optical amplifier; PD—optical photodetector; ADC—analog-to-digital converter; BEDFA—bidirectional optical erbium-doped fiber amplifier; PC—personal computer.

Figure 1 shows the schematic of the custom OTDR device. Sequential rectangular pulses with 34 GHz spectral bandwidth are generated by a 1530.6 nm laser diode (LD) controlled by an FPGA unit. These probing pulses are sent to port “1” of the optical circulator, followed by a 100 GHz optical bandpass filter that reduces the contribution of ASE noise. The pulses are amplified to 100 mW by the variable bidirectional optical amplifier (VAMP). The amplifier acts both as a booster for the OTDR pulses and as a preamplifier for the returned radiation measured by the 1 MHz photodetector (PD). The electrical signal from the detector is processed by the 156.25 MS/s analog-to-digital converter (ADC) connected to the computer.

### 2.2. Data Processing

**Figure 2:**
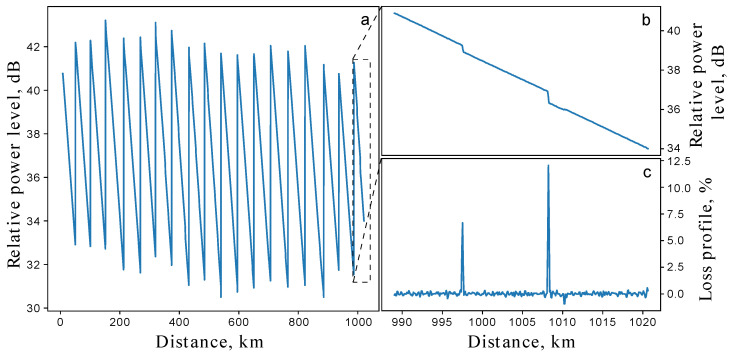
(**a**) Experimental OTDR trace of the entire 1020 km fiber line with 18 amplifiers. The measurements were performed with a probing pulse duration of 1 μs and averaging over 1.5 × 10^4^ pulses. (**b**) The reflectogram of the last fiber span. (**c**) The loss map of the last fiber span in percent scale.

Raw data from the ADC are compressed along the spatial axis to 80 m resolution to be in agreement with the bandwidth of the detector and probing pulse duration. The data are then processed into a typical dB-linear plot called a reflectogram. It provides information about all events in the optical fiber line-splices, bends, reflections, line damage, etc. Most reflectograms consist of a series of linear decays corresponding to the exponential decay of power in the medium. A sample of the OTDR trace of the presented line, recorded with probing pulses of 1 μs duration, can be seen in Figure 2a. Steep slopes correspond to optical amplifiers. Figure 2b shows the zoomed last fiber span in the optical line.

Before leakage detection, we additionally reduce the noise by using the $L_{1}$-filtering technique [38]. This is an optimisation problem that is formulated as

(1) $$\min_{x}{(∥Ax-y∥}_{2}^{2}+{\lambda ∥x∥}_{1}),$$

where the vector *y* is the original data and *x* is a vector of weights constrained by $L_{1}$ regularization with the hyperparameter λ. In our problem, the matrix *A* has the form

(2) $$A=\left[\begin{array}{ccccccc} 0 & 1 & 0 & 0 & \cdots & 0 & 0\\ 1 & 1 & 1 & 0 & \cdots & 0 & 0\\ 2 & 1 & 1 & 1 & \cdots & 0 & 0\\ \vdots & \vdots & \vdots & \; & \ddots & \vdots & \vdots \\ n-2 & 1 & 1 & 1 & \cdots & 1 & 0\\ n-1 & 1 & 1 & 1 & \cdots & 1 & 1 \end{array}\right].$$

This technique builds an approximation of the data as a general linear trend and a sparse weighted composition of step functions. The resulting solution Y=Ax is less noisy than the original data and forms the “filtered” reflectogram with a much smaller number of leakage candidates. After the $L_{1}$ filtering procedure, we extract a loss map LM, calculated as

(3) $$\mathrm{LM}=Y_{i}-Y_{i+t}+tx_{0},$$

where t=2 is chosen to deal with boundary effects of the discretization and $x_{0}$ denotes the general slope of the reflectogram. A loss map is a derivative-like plot that represents a series of peaks corresponding to stepwise losses in a trace. Using the peak detection method, we extract numerical evaluations of local leaks from the loss map and collect statistics. An example of a loss map is shown in Figure 2c.

## 3. Leakage Detection

In this section, we will demonstrate the limitations of OTDR-based monitoring on a long-haul transmission line. We use the same 1020 km fiber optic line. To detect a test leak at a point approximately 1009 km away, we modify the optical line by inserting an additional 2 km of fiber with access to its center. This is performed to have a separate test point that is far enough away from fiber connections or other events. We also measure this leakage independently using a direct measurement optical circuit. The most common, widely used, and easy-to-implement method for creating localized leakage is to bend the fiber. This is a very versatile approach because it can create losses ranging from fractions of a percent to those that result in almost complete cessation of light transmission. It is also a non-invasive method, there is no need to remove a protective polymer coating, and the optical fiber is not damaged between experiments. To implement this technique, we use semicircular grooves of different diameters to achieve a manageable change in loss.

**Figure 3:**
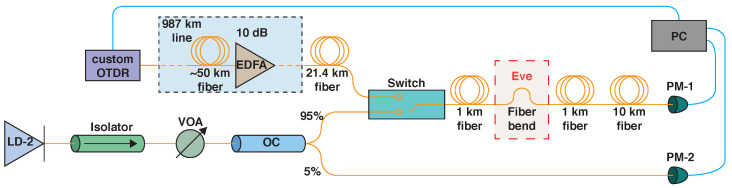
Scheme of the experimental setup with dual mode optical switch. EDFA—erbium-doped fiber amplifier; LD-2—laser diode; VOA—variable optical attenuator; OC—optical coupler; PM-1,2—optical powermeters; PC—personal computer.

To eliminate additional measurement errors, we do not remove the 2 km fiber span from the line during the direct measurement phase but instead use an optical switch-based setup with the scheme shown in Figure 3.

The switch toggles between the transmission coefficient measurement mode and the OTDR mode. A highly stable CWDM DFB laser diode (LD-2) with a central wavelength of 1533.1 nm and a bandwidth of approximately 1 MHz is used as the continuous light source. The variable optical attenuator (VOA) is installed to reduce optical power to 1 mW to prevent the development of stray Brillouin scattering in the optical fiber.

Two power meters (PM-1,2) are installed at the end of the line and at the lower connection of the 95/5 coupler (OC). When the switch is in transmission mode, data from both power meters are recorded. The resulting transmission loss coefficient is naturally calculated as $L_{T}=1-\frac{T_{\mathrm{bend}}}{T_{\mathrm{free}}}=1-\frac{\langle P_{1,\mathrm{bend}}\rangle }{\langle P_{2,\mathrm{bend}}\rangle }\cdot \frac{\langle P_{2,\mathrm{free}}\rangle }{\langle P_{1,\mathrm{free}}\rangle }$, where 〈⋯〉 denotes averaging over a time interval τ=60 s.

During the OTDR stage, measurements are made with a probing pulse duration of 1 μs and averaging over approximately $1.5\times 10^{4}$ pulses. Each measurement takes about 3 min. The reflectometric loss coefficient $L_{R}$ is taken from the resulting loss map as a peak value near the detected loss position and converted from the dB scale to the linear scale. It can then be further compared with $L_{T}$.

## 4. Results

### 4.1. Detection Without Reference

**Figure 4:**
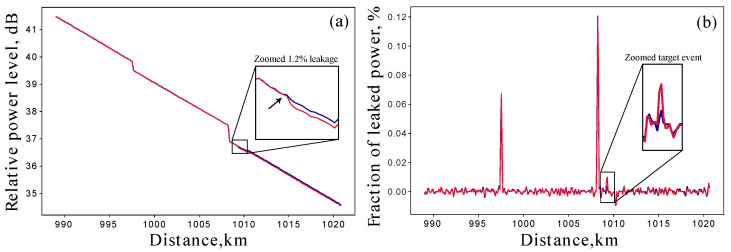
(**a**) OTDR traces of the last span of the line. The blue trace is the reference trace as in Figure 2b and the red trace is the trace of the line with the introduced 1.2% leakage at the position near 1009 km. (**b**) Corresponding loss maps of the two OTDR traces.

This section presents the results of test leakage experiments. Different leak detection scenarios have different limitations on the accuracy of detection. The most general case is the single unconditional measurement, where we have no prior information about the line to compare with. Figure 4a shows the reflectometry traces of the last span of the line with and without the 1.2% leak. The two images are shown for visual clarity and to clearly distinguish between the two measurements. The first drop at 997 km is the splice loss and the significant drop just after 1008 km is the switch loss. It can be seen that while the loss of more than 10% of the signal is clearly visible to the naked eye, the small loss of less than 1% is barely visible and requires some additional processing to observe.

Figure 4b shows the corresponding loss maps of the presented traces. It can be seen that there are two clearly visible loss events corresponding to a splice and the switch. In real application scenarios, they will also be counted as valid losses, but we will not take them into account as they are due to the experimental design. As $L_{R}$, we take the value of the threshold detected peak on the red plot. In our experiment, $L_{R}$ is estimated to be (1.16±0.06)% over the series of 20 measurements. The corresponding direct measurement estimate is $L_{T}=\left(1.20\pm 0.04\right)\%$.

To be consistent with the introduced value of $r_{E}$, which is not treated as a local lost fraction but as a lost fraction of the initial transmission, we should renormalize $L_{R}$ to the input pulse energy. With a ratio of 5.1 dB between the input energy and the current energy, we obtain $r_{E}=\left(0.39\pm 0.02\right)\%$. This operation can only be performed if the location of the leakage is known, e.g., by an OTDR approach in our case.

The fluctuations around the bottom of the loss map are not noise, but reproducible unique scattering features of the particular fiber span [39]. Their properties, such as spatial periods or amplitudes, depend drastically on the particular implementation of the reflectometry system: fiber type, operating wavelength, detector bandwidth, pulse width, and mathematical processing all contribute to the final image of these scattering characteristics. These features, estimated in our experiment to have a standard deviation of about $6.6\times 10^{-3}$ dB or $1.5\times 10^{-3}$, limit the referenceless detection approach. Thus, to achieve 6σ ($∼10^{-7}$ error probability) detection confidence with the current implementation, the estimated leakage cannot be less than 0.9%, which is a natural threshold for the approach. To overcome this limitation, reference traces, previously collected statistical data, or known physical properties of the fiber span should be used as additional data.

### 4.2. Detection with Reference

**Figure 5:**
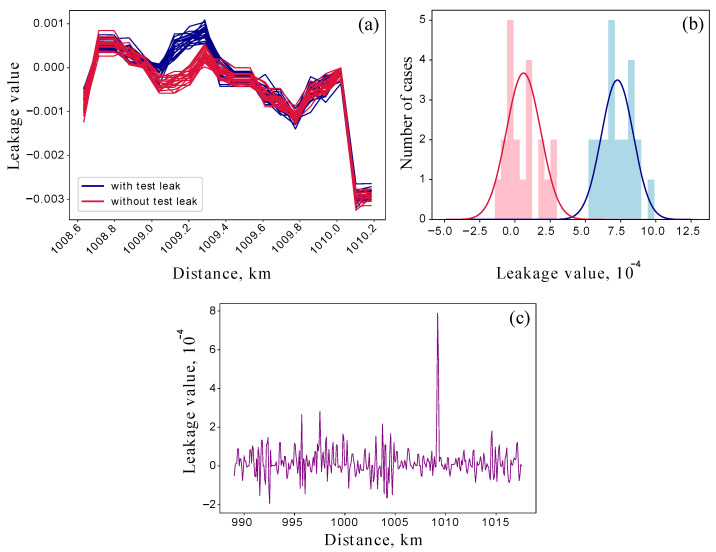
(**a**) Visualization of 2 series of OTDR measurements. The zoomed blue and red loss maps correspond to the line with and without the 0.2% test leakage. Such a small intrusion is hardly visible even on the loss map. (**b**) The statistics of the extracted losses for reference data (red histogram) and leakage data (blue histogram) approximated with the Gaussian distributions. (**c**) Visualization of the mean difference between reference and leakage maps. The statistics were taken over 400 pairs of traces. Although leakage does not match the estimate, it is statistically distinguishable and cannot be lost with a loss-negative result.

To get closer to the physical limits of the monitoring approach, we also performed an experiment that detects a test loss of about 0.2%. Estimation by direct measurement gives $L_{T}=\left(0.20\pm 0.04\right)\%$. Since such a leakage is comparable to the fluctuations in the lower level of the loss map, it is not easy to detect. Therefore, we obtained two sets of data: 20 reflectograms with the introduced leakage and 20 without. Figure 5a shows loss maps of the zoomed section near the intrusion point. The red plots are reference maps, while the blue plots correspond to 0.2% leakage. Figure 5b shows the statistical distributions of the extracted losses corresponding to the reference and leakage data. These statistics demonstrate the Gaussian distributions with the mean values of $\mu_{\mathrm{ref}}=0.6\times 10^{-4}$, $\mu_{\mathrm{leak}}=7.3\times 10^{-4}$ and the variances of $\sigma_{\mathrm{ref}}=1.2\times 10^{-4}$, $\sigma_{\mathrm{leak}}=1.1\times 10^{-4}$. It can be seen that they can be statistically distinguished with a probability of error of 0.25%. Figure 5c shows a pointwise mean difference between the reference and leakage maps. It can be seen that there is a very clear difference between the two sets of measurements. The error can be estimated knowing that the measurement result is the difference of two experimental values both having an error of 0.06%. As a result, the obtained error value is 0.09%, so $L_{R}=\left(0.08\pm 0.09\right)\%$ Despite the fact that we were unable to estimate the leak value accurately, the presence of the leak was reliably detected. Therefore, we report a leak detection of 0.2% or 0.01 dB on a 1009 km fiber line. In addition, this method also allows to detect additional leakage introduced in the place of the trace where it was already present, for example, in the splice point.

### 4.3. Temporal Stability

**Figure 6:**
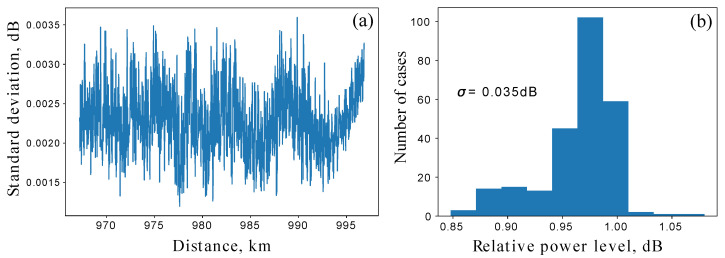
(**a**) The pointwise standard deviation of the reflectogram per day. This graph reflects its own drift for 1 day with a current implementation of the line and OTDR instrument itself. (**b**) The histogram showing the distribution of OTDR values at the intrusion point during the day. Its standard deviation is σ=0.035 dB, which corresponds to a variation of 0.8%.

To justify a reference-based approach, temporal stability measurements were performed. Specifically, a time series of 205 measurements over 25 h was obtained. Figure 6a shows a pointwise daily standard deviation of the OTDR trace and Figure 6b shows an OTDR value distribution at the leakage point.

The measurements show significant stability on a daily scale with a standard deviation not exceeding 0.8% along the fiber length.

## 5. Conclusions and Discussion

In this study, we have investigated the capabilities of conventional OTDR as a method for providing physical estimates of eavesdropper influence. The results presented in the Results section show that our OTDR-based approach can successfully identify local leaks over an unprecedented distance of 1009 km. In a single unconditional measurement scenario, we achieved 1.2% local leakage detection and explored the physical limits of this approach. In reference detection cases, we achieved detection of 0.2% local leakage, which is consistent with typical fusion splice losses in fiber optic lines. This level of accuracy is critical because it provides a basis for future improvements in leak detection methods. In addition, the effectiveness of certain protocols [15,20] may be highly sensitive to even small fractions of leakage.

In addition, our experiments explored the physical limits to detection imposed by the fundamental scattering properties of optical fibers. We provided insight into how these limitations can be addressed to achieve greater sensitivity in future applications. The monitoring approach we used is particularly relevant given the growing interest [12,13,14,15,20] in innovative techniques for quantum communication. The ability to detect even minimal leakage over such large distances allows QCKD systems to dynamically adapt or respond to threats in real time.

Looking ahead, the study of the system sensitivity on distance between the amplifiers is of interest to further research. Testing our approach in real field conditions on a deployed fiber-optic line, as well as the stability observed during the long-haul study, improving spatial resolution and measurement accuracy, is one of the development steps and can be the focus of a future paper. Moreover, the consideration of various eavesdropping attacks theoretically and practically is of interest. It is also an exciting issue to consider the integration of our solution with WDM data transmission systems.

## Data Availability

All materials that support the results of this study are available from the corresponding author upon reasonable request.

## References

[1] Renner R., Wolf R. (2023). Quantum Advantage in Cryptography. AIAA J..

[2] Pirandola S., Laurenza R., Ottaviani C., Banchi L. (2017). Fundamental limits of repeaterless quantum communications. Nat. Commun..

[3] Boaron A., Boso G., Rusca D., Vulliez C., Autebert C., Caloz M., Perrenoud M., Gras G., Bussières F., Li M.J. (2018). Secure Quantum Key Distribution over 421 km of Optical Fiber. Phys. Rev. Lett..

[4] Wang S., Chen W., Guo J.F., Yin Z.Q., Li H.W., Zhou Z., Guo G.C., Han Z.F. (2012). 2 GHz clock quantum key distribution over 260 km of standard telecom fiber. Opt. Lett..

[5] Korzh B., Lim C.C.W., Houlmann R., Gisin N., Li M.J., Nolan D., Sanguinetti B., Thew R., Zbinden H. (2015). Provably secure and practical quantum key distribution over 307 km of optical fibre. Nat. Photonics.

[6] Jouguet P., Kunz-Jacques S., Leverrier A., Grangier P., Diamanti E. (2013). Experimental demonstration of long-distance continuous-variable quantum key distribution. Nat. Photonics.

[7] Pan Y., Wang H., Shao Y., Pi Y., Li Y., Liu B., Huang W., Xu B. (2022). Experimental demonstration of high-rate discrete-modulated continuous-variable quantum key distribution system. Opt. Lett..

[8] Wang S., Yin Z.Q., He D.Y., Chen W., Wang R.Q., Ye P., Zhou Y., Fan-Yuan G.J., Wang F.X., Chen W. (2022). Twin-field quantum key distribution over 830-km fibre. Nat. Photonics.

[9] Liu Y., Zhang W.J., Jiang C., Chen J.P., Zhang C., Pan W.X., Ma D., Dong H., Xiong J.M., Zhang C.J. (2023). Experimental Twin-Field Quantum Key Distribution over 1000 km Fiber Distance. Phys. Rev. Lett..

[10] Simon C., De Riedmatten H., Afzelius M., Sangouard N., Zbinden H., Gisin N. (2007). Quantum repeaters with photon pair sources and multimode memories. Phys. Rev. Lett..

[11] Chen Y.A., Zhang Q., Chen T.Y., Cai W.Q., Liao S.K., Zhang J., Chen K., Yin J., Ren J.G., Chen Z. (2021). An integrated space-to-ground quantum communication network over 4,600 kilometres. Nature.

[12] Kirsanov N.S., Pastushenko V.A., Kodukhov A.D., Yarovikov M.V., Sagingalieva A.B., Kronberg D.A., Pflitsch M., Vinokur V.M. (2023). Forty thousand kilometers under quantum protection. Sci. Rep..

[13] Popp A., Sedlmeir F., Stiller B., Marquardt C. (2024). Eavesdropper localization for quantum and classical channels via nonlinear scattering. Opt. Quantum.

[14] Sushchev I.S., Bulavkin D.S., Bugai K.E., Sidelnikova A.S., Dvoretskiy D.A. (2024). Trojan-horse attack on a real-world quantum key distribution system: Theoretical and experimental security analysis. Phys. Rev. Appl..

[15] Kodukhov A.D., Pastushenko V.A., Kirsanov N.S., Kronberg D.A., Pflitsch M., Vinokur V.M. (2023). Boosting Quantum Key Distribution via the End-to-End Loss Control. Cryptography.

[16] Long G.L., Liu X.S. (2002). Theoretically efficient high-capacity quantum-key-distribution scheme. Phys. Rev. A.

[17] Pan D., Long G., Yin L., Sheng Y.B., Ruan D., Ng S., Lu J., Hanzo L. (2024). The Evolution of Quantum Secure Direct Communication: On the Road to the Qinternet. IEEE Commun. Surv. Tutor..

[18] Zeng H., Du M.M., Zhong W., Zhou L., Sheng Y.B. (2023). High-capacity device-independent quantum secure direct communication based on hyper-encoding. Fundam. Res..

[19] Cao Z., Lu Y., Chai G., Yu H., Liang K., Wang L. (2023). Realization of Quantum Secure Direct Communication with Continuous Variable. Research.

[20] Kirsanov N., Pastushenko V., Kodukhov A., Aliev A., Yarovikov M., Strizhak D., Zarubin I., Smirnov A., Pflitsch M., Vinokur V. (2024). Loss Control-Based Key Distribution under Quantum Protection. Entropy.

[21] Barnoski M., Rourke M., Jensen S., Melville R. (1977). Optical time domain reflectometer. Appl. Opt..

[22] Tian X., Dang R., Tan D., Liu L., Wang H. 123 km *Φ*-OTDR system based on bidirectional erbium-doped fiber amplifier. Proceedings of the Optical Communication, Optical Fiber Sensors, and Optical Memories for Big Data Storage.

[23] Wang Z., Zeng J., Li J., Peng F., Zhang L., Zhou Y., Wu H., Rao Y. 175 km phase-sensitive OTDR with hybrid distributed amplification. Proceedings of the 23rd International Conference on Optical Fibre Sensors.

[24] Wang Y., Wang Y., He C., Liu X., Bai Q., Jin B. (2023). 190 km *Φ*-OTDR with bidirectional Raman and relay erbium-doped fiber hybrid amplification. Opt. Lasers Eng..

[25] Fan C., Li H., Zhang K., Liu H., Sun Y., Liu H., Yan B., Yan Z., Liu D., Shum P.P. (2023). 300 km ultralong fiber optic DAS system based on optimally designed bidirectional EDFA relays. Photonics Res..

[26] Sumida M., Imai T., Furukawa S.i. Fault location on optical amplifier submarine systems. Proceedings of the Conference Proceedings. 10th Anniversary. IMTC/94. Advanced Technologies in I & M. 1994 IEEE Instrumentation and Measurement Technolgy Conference (Cat. No. 94CH3424-9).

[27] Furukawa S.i., Tanaka K., Koyamada Y., Sumida M. (1995). Enhanced coherent OTDR for long span optical transmission lines containing optical fiber amplifiers. IEEE Photonics Technol. Lett..

[28] Sumida M. (1996). Optical time domain reflectometry using an M-ary FSK probe and coherent detection. J. Lightwave Technol..

[29] Otani T., Horiuchi Y., Kawazawa T., Goto K., Akiba S. (1998). Fault localization of optical WDM submarine cable networks using coherent-optical time-domain reflectometry. IEEE Photonics Technol. Lett..

[30] Iida H., Toge K., Ito F. Environmental perturbation tracking in coherent OTDR for recovering detection sensitivity. Proceedings of the 39th European Conference and Exhibition on Optical Communication (ECOC 2013).

[31] Chen M., Zhang M., Chen S., Zhang J., Yan S., Wang Y. (2020). Health monitoring of long-haul fiber communication system using chaotic OTDR. China Commun..

[32] Fernández M.P., Rossini L.A.B., Pascual J.P., Caso P.A.C. (2018). Enhanced fault characterization by using a conventional OTDR and DSP techniques. Opt. Express.

[33] Rizzo A., Magri L., Rutigliano D., Invernizzi P., Sozio E., Alippi C., Binetti S., Boracchi G. (2022). Known and unknown event detection in OTDR traces by deep learning networks. Neural Comput. Appl..

[34] Abdelli K., Grießer H., Tropschug C., Pachnicke S. (2022). Optical Fiber Fault Detection and Localization in a Noisy OTDR Trace Based on Denoising Convolutional Autoencoder and Bidirectional Long Short-Term Memory. J. Lightwave Technol..

[35] Li Y., Zhang D., Wang Z., Yang H., Yu T., Yao Q., Liu S., Wang D., Zhao Y., Li H. (2023). Field trial of concurrent co-cable and co-trench optical fiber online identification based on ensemble learning. Opt. Express.

[36] Burdin V.A., Bourdine A.V., Zaitseva E.S., Praporshchikov D.E., Andreev V.A., Bourdine A.V., Burdin V.A., Morozov O.G., Sultanov A.C. (2021). Application of MIMO concept to detect nonreflecting events on cable fiber traces. Proceedings of the Optical Technologies for Telecommunications 2020.

[37] Desurvire E., Bayart D., Desthieux B., Bigo S. (2002). Erbium-Doped Fiber Amplifiers: Device and System Developments.

[38] Kim S.J., Koh K., Boyd S., Gorinevsky D. (2009). *ℓ*_1_ trend filtering. SIAM Rev..

[39] Smirnov A., Yarovikov M., Zhdanova E., Gutor A., Vyatkin M. (2023). An Optical-Fiber-Based Key for Remote Authentication of Users and Optical Fiber Lines. Sensors.

